# Modular and Distributed Supervisory Control Framework for Intelligent Micro-Manufacturing Systems with Unreliable Events

**DOI:** 10.3390/mi16101076

**Published:** 2025-09-23

**Authors:** Gaosen Dong, Zhengfeng Ming, Hesuan Hu

**Affiliations:** School of Electro-Mechanical Engineering, Xidian University, Xi’an 710071, China

**Keywords:** intelligent micro-manufacturing systems, industrial information integration, distributed supervisory control, robustness classification, detector automaton

## Abstract

This paper presents a modular and distributed supervisory control integration framework for intelligent micro-manufacturing systems (MMSs) under event-level failures. Addressing the increasing demand for scalable and reliable supervisory control in both micro- and smart manufacturing, the proposed approach equips each subsystem with a detector automaton that classifies runtime states into Strictly robust, Recoverably robust, or Non-robust categories. Distributed supervisors then make real-time local decisions to ensure fault-tolerant evolution of system behaviors. Unlike conventional centralized or Petri net-based methods, the proposed automaton-based framework supports modular design and structural scalability. Quantitative comparisons show that the robustness-detection cost scales approximately linearly with the summed sizes of local graphs, indicating good structural scalability. Simulation studies validate the feasibility and scalability of the framework, demonstrating its effectiveness in maintaining production cycle reachability and its integration potential for micro-electro-mechanical systems (MEMS)-based production lines, micro-fabrication platforms, and smart factory environments. These results confirm that the proposed method can serve as a robust and deployable control layer for next-generation intelligent and micro-manufacturing integration architectures.

## 1. Introduction

With the rapid advancement of industrial information integration and the emergence of smart and micro-manufacturing paradigms, there is a critical need for scalable and robust supervisory control frameworks in automated manufacturing systems (AMSs). Modern AMSs increasingly require seamless integration between event-driven supervisory logic and industrial information systems, edge computing platforms, and smart devices to ensure safe, adaptive, and resilient operation under uncertainty. This requirement is particularly critical for micro-fabrication and MEMS production, where process outcomes are highly sensitive to dynamic conditions (e.g., surface roughness and feature/yield variability) [1,2]. While recent data-driven approaches can predict quality indicators or optimize yield, they do not provide a formal, verifiable supervisory logic to govern how the system should react under uncertainty [1,2]. Our work addresses this gap by proposing a distributed, automaton-based supervisory framework.

Supervisory control, originally proposed by Ramadge and Wonham [3], has become a key method to restrict the behavior of discrete-event systems (DESs) by enabling or disabling controllable events to enforce desired specifications. Unlike traditional feedback control in continuous systems, supervisory control offers a formal, event-driven framework that has been widely applied to industrial scenarios such as manufacturing [4], traffic management [5], communication protocols [6], and robotic coordination [7].

The theoretical foundations of supervisory control are built upon the notions of controllability, observability, and nonblockingness. Over time, these foundations have been extended to support critical features such as fault diagnosis [8,9,10], detectability [11,12,13], opacity [14,15,16], communication delays [17,18], and attack modeling and resilience [19,20,21]. These extensions aim to enhance the observability, security, and robustness of DESs in uncertain environments, which is essential in domains such as smart manufacturing, cyber–physical systems, and critical infrastructure.

Such robustness concerns are especially critical in smart and micro-manufacturing systems, including MEMS-integrated production lines, where unreliable events and subsystem failures may lead to safety violations or task incompleteness, which motivates the development of robust and distributed supervisory control frameworks capable of handling such uncertainties. Among the various application domains, automated manufacturing systems (AMSs) represent one of the most representative real-world implementations of DES theory. In practice, AMSs frequently encounter uncertainties including event failures, communication latency, sensor faults, and external disturbances, which may cause system behaviors to deviate from their intended trajectories, potentially resulting in safety violations or degraded performance. To mitigate such risks, robust supervisory control in AMSs has attracted increasing research interest, with the objective of ensuring that core behaviors, task reachability, and nonblockingness are preserved even in the presence of local anomalies, structural perturbations, or environmental variations [22,23,24,25,26,27,28,29,30].

Much of the existing literature on robustness in AMSs is based on Petri net modeling. For instance, [25,29] introduced the concept of maximal perfect resource-transition circuits (MPC) to characterize blocking states caused by resource failures. Other studies, such as [26], proposed synthesis methods using strong covering structures or saturated siphon constructs to design robust supervisors. Reachability graph-based methods for robustness analysis have recently become mainstream [24,27,28,30].

Despite the progress achieved in Petri net-based AMS robustness, most of these studies rely on centralized control architectures. Few unified frameworks exist that adopt finite automata as the modeling basis while integrating unreliable event modeling, robustness classification, distributed detector synthesis, and local enforcement into a cohesive control strategy. Compared with Petri nets, automaton-based modeling offers a more direct semantic alignment with supervisory control logic, better supports modular composition, and simplifies detector construction and implementation, which is particularly important for MMSs that are highly sensitive to event failures and resource perturbations.

To address this gap, this paper proposes a distributed detector-based robust supervisory control framework specifically designed for AMSs subject to unreliable events and partial observation. The proposed method supports runtime robustness classification and localized control decision-making while preserving the global system’s safety and liveness properties.

Unlike previous studies that focus on Petri net-based robustness [24,27,28,30], this work builds a detector-based framework under automaton semantics, which simplifies classification logic and supports distributed enforcement without global synthesis. Compared with existing Petri net-based robustness control methods [29,30], the proposed automaton-based approach offers the following benefits:

- Explicit state and transition structures better aligned with supervisory control logic;
- Easier construction of local detectors via synchronous composition and projection;
- Avoidance of state explosion during reachability graph enumeration.

Moreover, unlike traditional Petri net-based methods, the proposed automaton-based strategy offers not only formal clarity and modular implementation advantages but also better alignment with runtime deployment needs in intelligent MMS/MEMS platforms, and it simplifies the construction and deployment of detector automata, which is particularly important for AMSs that are highly sensitive to event failures and resource perturbations.

In the industrial context, modern micro-manufacturing cells (e.g., laser micromachining, micro-assembly/packaging, and micro-inspection) are organized as small DES subsystems that share resources such as vacuum pumps, precision stages, grippers, and inspection microscopes. Because parts and wafers are fragile and tolerances are tight, these cells are highly sensitive to event-level faults (loss of vacuum, misalignment, tool jam, vision rejection). Our framework fits this context for several reasons: (i) Event-level enforcement: robustness is decided at the event level, matching the granularity of the above faults. (ii) Local real-time decisions: supervisors inspect detector labels over the local event alphabet and a small set of enabled events, enabling millisecond-scale reactions on PLC/ROS without global search. (iii) Modularity in production cells: the detector-based design isolates faults locally and supports incremental cell integration across reconfigurable lines.

The main contributions of this paper include the following:

- A modular and distributed supervisory control integration framework is proposed for AMSs/MMSs, supporting robust execution under event failures and seamless integration into industrial information systems without requiring centralized coordination or global model construction.
- Local detector automata are designed for each subsystem to classify operational states into Strictly robust, Recoverably robust, or Non-robust categories, thereby enabling real-time decision-making, enhanced information integration, and local control based on observed data streams.
- The proposed control strategy offers a scalable and adaptable industrial solution that avoids unsafe trajectories, supports controller reusability and system extensibility, and is compatible with deployment on PLC-based, edge computing, and industrial information management platforms. It is also suitable for micro-manufacturing execution systems and MEMS-oriented production platforms.

The remainder of this paper is organized as follows. Section 2 introduces the modeling framework and defines event unreliability. Section 3 presents the problem formulation and robustness classification. Section 4 details the construction of detectors and the distributed control strategy. Section 5 provides simulation validation. Section 6 concludes this paper.

## 2. Preliminaries and Modeling for Automated and Micro-Manufacturing Systems

This section introduces basic definitions and notations used throughout the paper. We establish a unified modeling framework based on modular DESs, define relevant language operations, and introduce event classifications that will be used to formally express robustness concepts.

### 2.1. Modular System Model

We consider an AMS/MMS composed of *N* local modules. Each module is modeled as a deterministic finite-state automaton:

$$G_{i}=\left(X_{i},E_{i},f_{i},\Gamma_{i},x_{0,i}\right),\;i=1,\ldots ,N,$$

where $X_{i}$ is the state set, $E_{i}$ the event set, $f_{i}$ the transition function, $\Gamma_{i}$ the set of enabled events, and $x_{0,i}$ the initial state. The global system is given by synchronous composition:

$$G={\|}_{i=1}^{N}G_{i}=\left(Q,E,f,\Gamma ,Q_{0}\right),$$

with $Q=\prod_{i=1}^{N}X_{i}$, $E={⋃}_{i=1}^{N}E_{i}$, and $Q_{0}=\left(x_{0,1},\ldots ,x_{0,N}\right)$. Synchronization occurs over shared events.

### 2.2. Event Classification

The global event set *E* is classified as follows:

- Control-based: $E=E_{c}\cup E_{uc}$, where $E_{c}$ denotes the set of controllable events and $E_{uc}$ denotes the set of uncontrollable events.
- Observation-based: $E=E_{o}\cup E_{uo}$, where $E_{o}$ denotes the set of observable events and $E_{uo}$ denotes the set of unobservable events.
- Reliability-based: $E=E_{r}\cup E_{ur}$, where $E_{r}$ denotes the set of reliable events and $E_{ur}$ denotes the set of unreliable events.

Each local supervisor $S_{i}$ only observes $E_{i}^{o}\subseteq E_{i}$ and controls $E_{i}^{c}\subseteq E_{i}\setminus E_{uc}$.

### 2.3. Language and Projections

Let $E^{*}$ denote the set of all finite event sequences. The language generated by *G* is

$$L\left(G\right)=\{s\in E^{*}|f\left(x_{0},s\right)!\}.$$

Other useful constructs include the following:

- Prefix closure: $\bar{L(G)}=\left\{s\in E^{*}|\exists t\in E^{*},st\in L\left(G\right)\right\}$;
- Post-language: $L\left(G\right)/s=\{t\in E^{*}|st\in L\left(G\right)\}$;
- Projection: For $E_{i}^{o}\subseteq E$, define $P_{i}:E^{*}\to E_{i}^{o*}$, erasing events not in $E_{i}^{o}$.

### 2.4. Robustness-Related Language Sets

To characterize system robustness, we define two special subsets:

The first is the unreliable string set:

$$E_{ur}^{+}=\left\{s\in E^{*}|\exists e\in E_{ur},e\in s\right\},$$

which includes all strings containing at least one unreliable event.

The second is the terminal language $L_{end}$.

For each $G_{i}$, we define a set of semantically meaningful terminal states $X_{i}^{end}\subseteq X_{i}$, representing successful completion of production cycles. These states are declared based on the model’s physical context (e.g., reaching the last processing step of a product).

The global terminal state set is

$$X^{end}=\prod_{i=1}^{N}X_{i}^{end},$$

and the terminal language is

$$L_{end}=\left\{s\in L\left(G\right)|f\left(x_{0},s\right)\in X^{end}\right\}.$$

### 2.5. Illustrative Example: A Structured AMS Model

#### 2.5.1. AMS Layout and Structural Motivation

**Figure 1:**
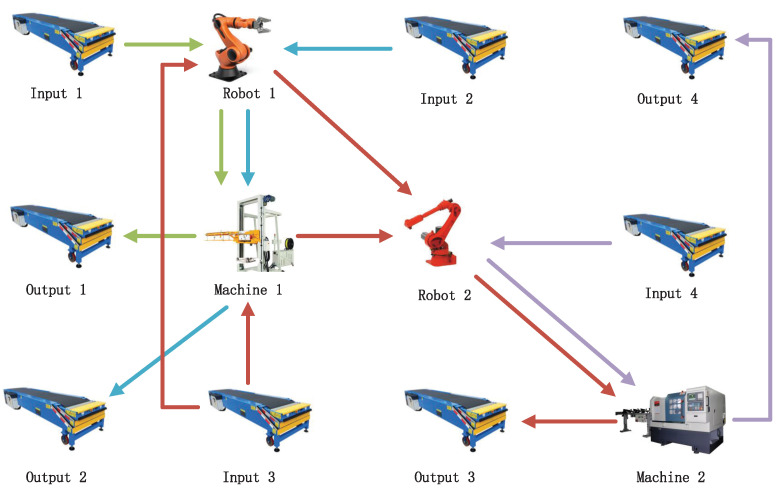
Illustrative AMS layout including input/output conveyors, robotic arms, and machines. Arrows show material flows and resource-coordination links; arrow colors are only for visual distinction of different paths and do not encode additional semantics.

To provide physical insight into our modeling framework, we begin with a realistic layout of an AMS as shown in Figure 1. The system consists of four input/output conveyor lines, two industrial robots, and two CNC-type machines. The layout captures typical component interactions and material transport paths in a real-world AMS.

Based on this structure, we now abstract the behavior of each subsystem using modular finite-state automata, as described below.

#### 2.5.2. Product Automata

**Figure 2:**
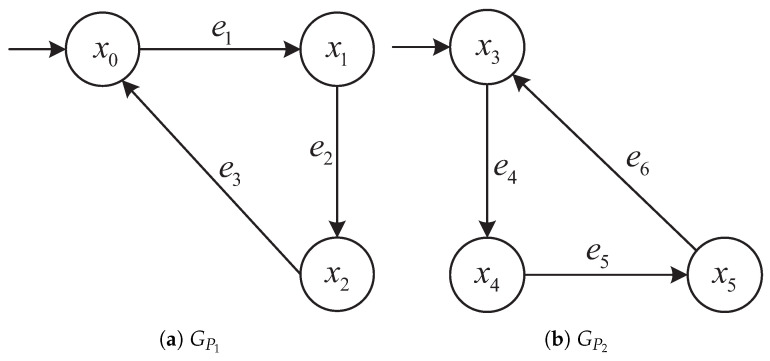

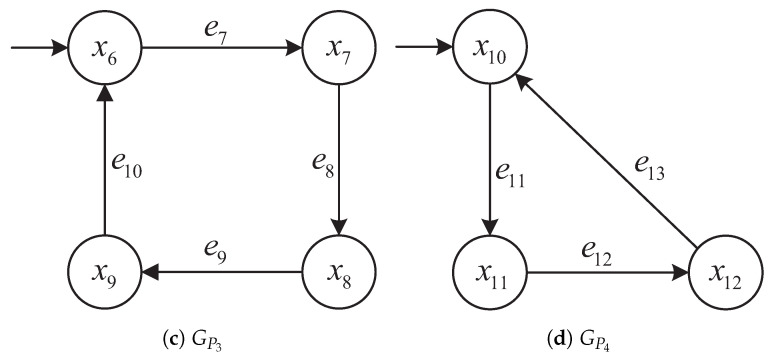
Product automata $G_{P_{1}}$ to $G_{P_{4}}$ with terminal states $x_{0}$, $x_{3}$, $x_{6}$, $x_{10}$.

Each product automaton $G_{P_{i}}=\left(X_{P_{i}},E_{P_{i}},f_{P_{i}},x_{0,P_{i}}\right)$ describes the flow of a specific product type. Figure 2 shows the four product automata $G_{P_{1}},G_{P_{2}},G_{P_{3}},G_{P_{4}}$, respectively. Transitions represent production steps; certain transitions such as $e_{3}$, $e_{6}$, $e_{10}$, and $e_{13}$ correspond to final product completion steps and return the system to the initial states $x_{0}$, $x_{3}$, $x_{6}$, and $x_{10}$, respectively. These states serve as terminal states in our modeling, indicating the completion of a full production cycle and readiness for the next.

#### 2.5.3. Resource Automata

**Figure 3:**
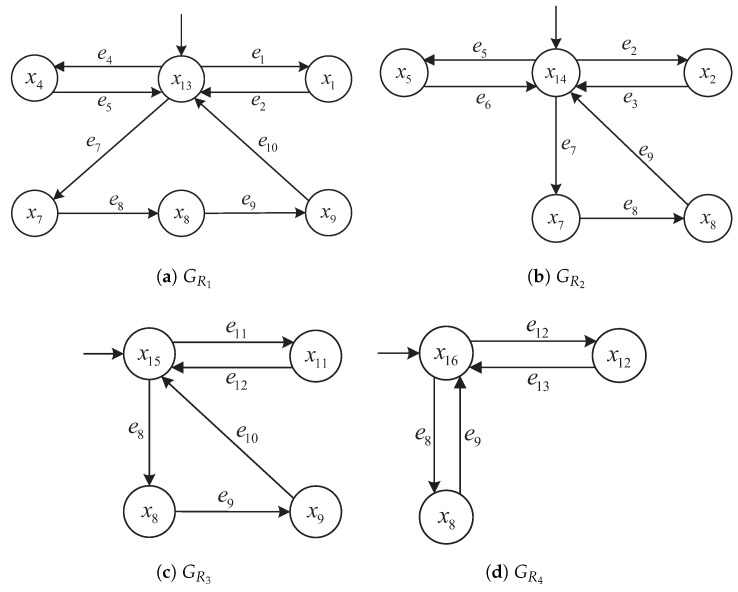
Resource automata $G_{R_{1}}$ to $G_{R_{4}}$ showing synchronization with product events.

Each resource automaton $G_{R_{j}}=\left(X_{R_{j}},E_{R_{j}},f_{R_{j}},x_{0,R_{j}}\right)$ models the operational status of a shared resource (e.g., machine, buffer, workstation). Figure 3 shows four resource automata $G_{R_{1}}$ to $G_{R_{4}}$ that synchronize with corresponding product transitions to coordinate resource usage. For example, events $e_{5}$, $e_{8}$, $e_{10}$, and $e_{13}$ appear in both product and resource automata to represent shared transitions.

#### 2.5.4. Global Synchronous System

The entire AMS is modeled as the synchronous composition

$${G=\|}_{i=1}^{4}G_{P_{i}}{\left\|\right\|}_{j=1}^{4}G_{R_{j}}.$$

Here, each synchronization occurs over the intersection of shared events, such as $e_{3}$, $e_{6}$, $e_{10}$, and $e_{13}$, which represent jointly executed operations between product lines and corresponding resources. The transition structure of *G* encodes all inter-module dependencies and provides the operational foundation for distributed supervision.

**Figure 4:**
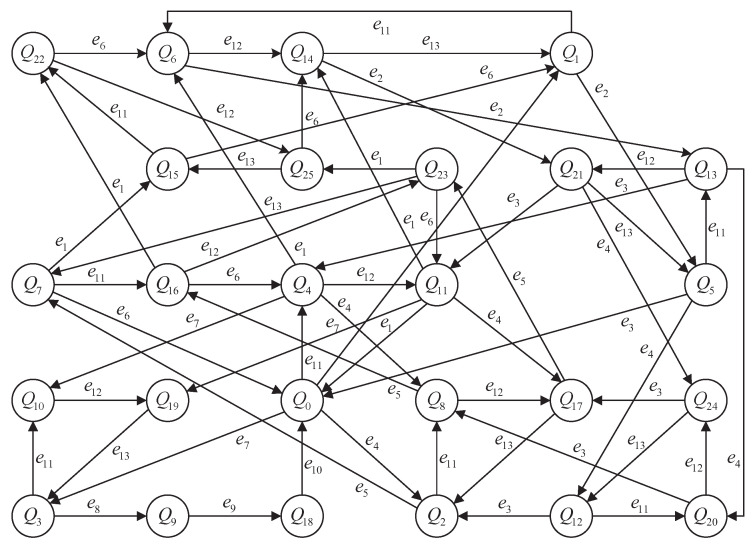
Global AMS synchronization structure.

Figure 4 illustrates the global synchronization structure of the AMS, showing interactions among all product and resource modules via shared events.

In practice, due to the state explosion of full synchronization, we analyze subsystems (e.g., $G_{P_{1}}\|\;G_{R_{1}}\|\;G_{P_{2}}$) in subsequent sections to demonstrate key concepts such as detector construction and robust supervisory control.

Each final event $e_{3}$, $e_{6}$, $e_{10}$, and $e_{13}$ leads the system back to its initial states, indicating a completed processing cycle. Therefore, we define the terminal state set as

$$Q^{end}=\left\{\left(x_{P},x_{R}\right)\in \left(\Pi_{i}^{4}X_{P_{i}}\right)\times \left(\Pi_{j}^{4}X_{R_{j}}\right)|\exists i\in \left\{1,\ldots ,4\right\}:x_{P_{i}}=x_{0,P_{i}}\right\}$$

Rationale: In our AMS model, each terminal product event $\left(e_{3},e_{6},e_{10},e_{13}\right)$ synchronizes with resource-release transitions that return the involved resources to their idle nodes (see Figure 3). Hence, no explicit constraint on the resource component of $\left(x_{P},x_{R}\right)$ is required.

The terminal language is then

$$L_{end}=\left\{s\in L\left(G\right)|f\left(Q_{0},s\right)\in Q^{end}\right\}.$$

#### 2.5.5. Unreliable Events and Resource Failures

In this AMS framework, unreliable events represent potential failures occurring during interactions with shared resources. We focus specifically on faults related to the operation of resources $G_{R_{3}}$ and $G_{R_{4}}$.

Let $E_{ur}\subseteq E$ denote the set of unreliable events. We define the following:

- $E_{ur}=\left\{e_{9},e_{10},e_{12},e_{13}\right\}$.

These events represent transitions in product automata that synchronize with resource automata $G_{R_{3}}$ and $G_{R_{4}}$. In this context, failures in these resources (e.g., machine breakdowns or unavailable capacity) are modeled by assuming the associated events may be disabled due to resource-side faults such as breakdowns or unavailability. Supervisory strategies developed in later sections will aim to mitigate the risks posed by these unreliable events.

**Definition**  **1.** *An event e∈E is called unreliable if it is shared with a faulty resource automaton and may fail to be enabled due to unexpected faults. The collection of such events is denoted by $E_{ur}$.*

**Proposition**  **1.** *If $e\in E_{ur}$ is an unreliable event, then under fault conditions, e is not guaranteed to be enabled in the global system G even if all product-side conditions are satisfied.*

**Proof.**  Since *G* is the synchronous composition of product and resource automata, event $e\in E_{ur}$ is enabled in *G* at a global state $Q=(x_{P},x_{R})$ only if it is enabled in both the product component $G_{P}$ and the corresponding resource component $G_{R}$. If $G_{R}$ experiences a fault that disables *e* (e.g., resource is busy, failed, or unavailable), then *e* is not enabled in *G* regardless of the status of $G_{P}$. Thus, unreliability at the resource level can directly disable the execution of *e* in the global model.    □

This reflects that only strings leading to complete and ready-for-restart configurations are considered safe terminations.

## 3. Problem Formulation for Distributed Robust Supervision

### 3.1. Problem Formulation

Let $G=(Q,E,f,Q_{0})$ be the global plant defined in Section 2, where *E* is already partitioned into

$$E=E_{c}\cup E_{uc},\;E=E_{o}\cup E_{uo},\;E=E_{r}\cup E_{ur}.$$

Each local supervisor $S_{i}$ is defined over a partial alphabet $E_{i}\subseteq E$ and observes a subset $E_{i}^{o}\subseteq E_{i}$ and controls $E_{i}^{c}\subseteq E_{i}\cap E_{c}$.

Define the local observation projection:

$$P_{i}:E^{*}\to E_{i}^{o*},$$

which erases events not in $E_{i}^{o}$.

The local supervisor is a map:

$$S_{i}:E_{i}^{o*}\to 2^{E_{i}^{c}},$$

and the joint distributed supervisor is

$$S\left(s\right)=\overset{N}{\underset{i=1}{⋂}}S_{i}\left(P_{i}\left(s\right)\right).$$

Then the controlled behavior of the system is

$$L\left(S/G\right)=\{s\in L\left(G\right)|\forall k\leq |s|,s_{k}\in S\left(s_{<k}\right)\cup E_{uc}\}.$$

**Assumption** **1.** *(Full controllability and observability): Although the global event set includes uncontrollable ($E_{uc}$) and unobservable ($E_{uo}$) events, we assume that all locally relevant events are controllable and observable, i.e., $E_{c}^{i}=E_{i}$, $E_{o}^{i}=E_{i}$. This assumption enables the focus to remain on robustness enforcement under unreliable events while avoiding complications due to uncontrollability or unobservability. The extension to partial observation and limited control authority will be pursued in future work.*

Rationale: We adopt full controllability and observability locally to isolate the effect of unreliable events on robustness detection. This matches many PLC/ROS-based AMS cells where event execution and sensing at the cell level are fully actuated and instrumented; the treatment of limited control/partial observation is deferred to future work.

### 3.2. Distributed Robustness Criteria

We consider two types of robustness objectives:

**Definition** **2.** *The system is Strictly robust under S if*

$$L\left(S/G\right)\cap E_{ur}^{+}=\emptyset ,$$

*that is, no string executed under control contains any unreliable event.*

**Definition** **3.** *The system is Recoverably robust under S if*

$$\forall s\in \bar{L(S/G)},\exists s_{1}\in {\left(E\setminus E_{ur}\right)}^{*}\;such\;that\;ss_{1}\in L_{end}.$$

*That is, for every prefix of the controlled behavior, there exists a continuation string without any unreliable events that drives the system into a terminal state.*

**Definition** **4.** *Given a set of subsystems $\left\{G_{i}\right\}$ with individual detector automata $\left\{D_{i}\right\}$, we say the system is distributedly robust with respect to event failures in $E_{ur}$ if and only if the following hold: (i) the local robustness labels of $D_{i}$ are sound and complete with respect to the local behavior of $G_{i}$; and (ii) the composition of local robustness classifications under the merging rule $\phi_{global}$ ensures global nonblocking and task reachability.*

These robustness criteria reflect different tolerance levels to resource failures and will guide the synthesis of distributed supervisors in subsequent sections.

**Figure 5:**
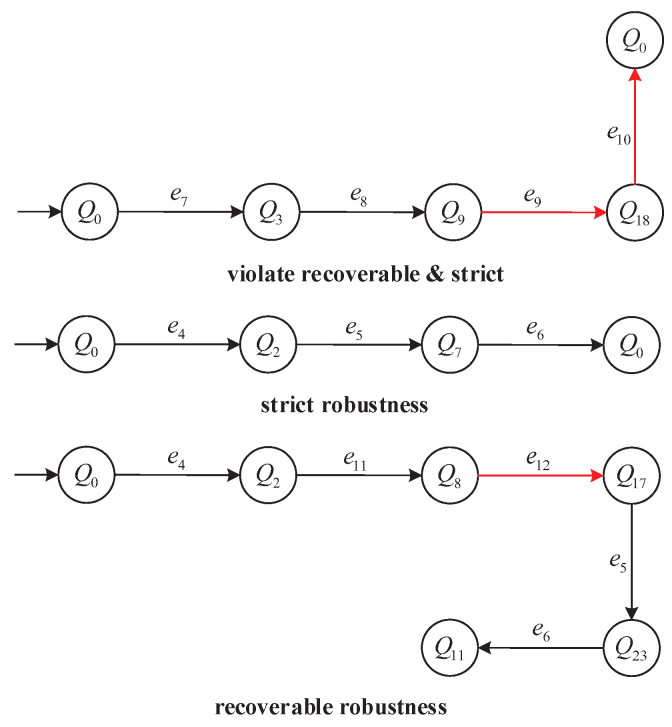
Execution-path classification in the AMS model. Red arcs indicate unreliable events $e\in E_{ur}$; black arcs indicate reliable events. Top path: not recoverable. Middle path: strict robustness. Bottom path: recoverable robustness.

To further clarify these robustness definitions, Figure 5 illustrates three representative execution paths from the AMS model *G*:

(i) Top path: Violates both Strict and Recoverable robustness, as it executes $e_{9}\in E_{ur}$ and no further continuation reaches a terminal state.

(ii) Middle path: Satisfies Strict robustness by using only reliable events and reaching a designated terminal state.

(iii) Bottom path: Satisfies Recoverable robustness by continuing through reliable events after $e_{12}\in E_{ur}$ to reach a terminal state.

**Lemma** **1.** *If s∈L(S/G) and s contains an event $e\in E_{ur}$, then the supervisor S cannot satisfy Strict robustness.*

**Proof.**  By definition of Strict robustness, the set L(S/G) must be disjoint from $E_{ur}^{+}$. If *s* contains any $e\in E_{ur}$, then $s\in E_{ur}^{+}$, thus violating $L\left(S/G\right)\cap E_{ur}^{+}=\emptyset$. Therefore, *S* fails to achieve Strict robustness.    □

The robustness distinctions illustrated above motivate a clear analytical foundation. The following lemma formally highlights the inherent limitation imposed by Strict robustness, which completely forbids occurrences of unreliable events.

### 3.3. Problem Statement

Problem: Given the global plant $G={∥}_{i=1}^{N}G_{i}$, local alphabets $\left(E_{i}^{o},E_{i}^{c}\right)$, and event partitions $\left(E_{ur},E_{uc},E_{uo}\right)$, synthesize distributed supervisors ${\left\{S_{i}\right\}}_{i=1}^{N}$ such that the resulting behavior satisfies Strict or Recoverable robustness.

Key technical challenges: Each $S_{i}$ only observes partial behaviors and controls local events. Ensuring that the global behavior

$$L_{global}=\overset{N}{\underset{i=1}{⋂}}P_{i}^{-1}\left(L\left(S_{i}/G_{i}\right)\right)$$

remains robust requires careful coordination under partial information.

Main conceptual challenges include the following:

- Limited local knowledge: Although all events are assumed to be locally observable and controllable in this work, real-world systems may include unobservable or uncontrollable events, which complicate supervision.
- Synchronization ambiguity: The execution of a shared event may depend on the state of another module that is not visible to the local supervisor.
- Fault propagation risk: A single unreliable event can propagate failures through multiple modules unless proactively prevented.

## 4. Distributed Robust Supervisor Design

To enable robust supervision under partial observation and unreliable events, this section proposes a distributed synthesis strategy that decomposes global analysis into localized decisions.

Centralized robust supervisory synthesis typically requires the explicit construction of the global plant ${G=\|}_{i=1}^{N}G_{i}$, which suffers from severe state explosion due to synchronous product operations. In the presence of unreliable events, the analysis of global robust reachability becomes even more intractable, as failure propagation must be tracked across all subsystems.

Centralized synthesis suffers from lack of modularity, as any subsystem update requires re-synthesizing the entire global model. This hinders scalability, adaptability, and practical implementation.

In contrast, the proposed distributed approach constructs local detectors $D_{i}$ and supervisors $S_{i}$ without requiring the global synchronous product, leveraging only local event structures and known unreliable events. This enables scalable synthesis, localized diagnosis, and runtime efficiency while still preserving global robustness guarantees through conservative decision fusion.

In systems composed of multiple interacting automata with local observations and possible event unreliability, centralized control methods often suffer from state explosion and lack of structural scalability. Specifically, the construction of the global plant $G=G_{1}\|G_{2}\left\|\cdots \right\|G_{N}$ and the corresponding monolithic supervisor becomes impractical as the number of subsystems increases or the event space becomes dense. (Throughout this paper, all events are observable and controllable; ‘local observations’ means each detector $D_{i}$ evolves on its own alphabet $E_{i}^{o}$, not that some events are unobservable.)

To overcome these limitations, we propose a distributed robustness framework that decomposes the control synthesis problem into localized robustness detection and enforcement tasks. The core idea is to endow each subsystem $G_{i}$ with a local detector $D_{i}$ that classifies its states into Strictly robust, Recoverably robust, or Non-robust according to whether reliable paths to local terminal states exist. These detectors are constructed using only the state space of $G_{i}$ and the known unreliable event set $E_{ur}$.

Formally, for each local observation history $s_{o}\in {\left(E_{i}^{o}\right)}^{*}$, the supervisor $S_{i}$ determines the current state $y\in Y_{D_{i}}$ of the detector and applies a conservative event-enablement rule:

$$S_{i}\left(s_{o}\right)=\left\{\begin{array}{cc} E_{i}^{c} & \mathrm{if}\;\varphi_{i}\left(y\right)=\mathrm{Strict},\\ E_{i}^{c}\setminus \left\{e\in E_{i}^{c}|e\to \mathrm{Non}\text{-}\mathrm{robust}\right\} & \mathrm{if}\;\varphi_{i}\left(y\right)=\mathrm{Recoverable},\\ \emptyset & \mathrm{if}\;\varphi_{i}\left(y\right)=\mathrm{Non}\text{-}\mathrm{robust}. \end{array}\right.$$

Unlike traditional methods, our framework ensures that supervisors avoid unsafe behaviors without global coordination or fault observability. The distributed strategy guarantees that the global language

$$L_{\mathrm{global}}=\overset{N}{\underset{i=1}{⋂}}P_{i}^{-1}\left(L\left(S_{i}/G_{i}\right)\right)$$

remains within the Strictly or Recoverably robust trajectories of the overall system, as proven in Theorem 1.

This design philosophy transforms the robustness problem from a centralized model-checking challenge into a modular, scalable synthesis approach, enabling large-scale implementation across fault-prone and information-constrained systems.

### 4.1. Distributed Robust State Detectors

The cornerstone of distributed robust supervision is the ability of local detectors to accurately classify the system states with respect to robustness. Distributed robust state detectors perform this function by analyzing local event sequences and predicting future execution outcomes.

#### Formal Definition and Construction

Formally, a local robust state detector associated with the local supervisor $S_{i}$ is defined as

$$D_{i}=\left(Y_{D_{i}},E_{i}^{o},f_{D_{i}},y_{D_{i}0},\varphi_{i}\right),$$

where

- $Y_{D_{i}}$ is the finite set of detector states;
- $E_{i}^{o}$ is the locally observable event set;
- $f_{D_{i}}:Y_{D_{i}}\times E_{i}^{o}\to Y_{D_{i}}$ is the transition function;
- $y_{D_{i}0}$ is the initial state of the detector;
- $\varphi_{i}:Y_{D_{i}}\to \left\{\mathrm{Strict},\mathrm{Recoverable},\mathrm{Non}\text{-}\mathrm{robust}\right\}$ is the robustness classification function.

Detectors evolve on the locally observable alphabet $E_{i}^{o}$, while the robustness classification only needs the reliable/unreliable split inside the local alphabet $E_{i}$ inherited from the global partition (see Step 3 below).

The construction method: is as follows:

1. Define each local subsystem as a combination of a product automaton and its relevant resource automata.
  $$G_{i}=G_{P_{i}}\|\left({\|}_{j\in J_{i}}G_{R_{j}}\right).$$
2. Track reachable states in the local subsystem based solely on local observable events.
3. Event partition: Globally, the event set is partitioned as $E=E_{r}⊎E_{ur}$ into reliable and unreliable events. For a local subsystem $G_{i}$ with alphabet $E_{i}\subseteq E$ and locally observable subset $E_{i}^{o}\subseteq E_{i}$, we inherit the global partition by intersection:
  $$E_{\mathrm{rel}}^{\left(i\right)}:=E_{i}\cap E_{r},\;E_{\mathrm{ur}}^{\left(i\right)}:=E_{i}\cap E_{ur},\;E_{i}\;=\;E_{\mathrm{rel}}^{\left(i\right)}⊎E_{\mathrm{ur}}^{\left(i\right)}.$$
  Events not in $E_{i}$ do not occur in $G_{i}$ and are irrelevant for local reasoning. The detector transitions use locally observable labels $E_{i}^{o}$, while robustness classification only needs the reliable/unreliable distinction inside $E_{i}$ as defined above. In Algorithm 1, we therefore operate on $G_{i}$ with two label sets: (i) $E_{i}$ when we compute forward reachability of *arbitrary* local prefixes, and (ii) $E_{\mathrm{rel}}^{\left(i\right)}$ when we require *reliable* prefixes or suffixes. Throughout the algorithm we write $E_{\mathrm{rel}}$ (resp. $E_{ur}$) for $E_{\mathrm{rel}}^{\left(i\right)}$ (resp. $E_{\mathrm{ur}}^{\left(i\right)}$) to simplify notation.
4. Classify states as follows:
  - Strict robustness: A state $y\in Y_{i}$ is Strict if there exist a reliable prefix $\sigma \in E_{\mathrm{rel}}^{*}$ from $Y_{i}^{0}$ to *y*, and there exists a reliable suffix $\tau \in E_{\mathrm{rel}}^{*}$ from *y* to a terminal state in $Y_{i}^{\mathrm{end}}$. Equivalently, *y* lies on a path that uses only reliable events up to *y* and can continue by reliable events to $Y_{i}^{\mathrm{end}}$.
  - Recoverable robustness: A state $y\in Y_{i}$ is Recoverable if there exists a path $s\in L(G_{i})$ from $Y_{i}^{0}$ to *y* such that every prefix $\bar{s}$ of *s* reaches a state that admits a reliable suffix to $Y_{i}^{\mathrm{end}}$. Hence, Strict and Recoverable share the same ‘reliable-suffix-to-terminal’ property; furthermore, Strict requires a reliable prefix, while Recoverable allows unreliable prefixes whose every prefix remains Recoverable by some reliable suffix.
  - Non-robust: A state not reachable to $Y^{\mathrm{end}}$ under reliable events.

Robust State Classification Algorithm 1 follows below.
**Algorithm 1** Robust State Classification with Prefix–Suffix Semantics.**Require:** Local automaton $G_{i}=\left(Y_{i},E_{i},\to \right)$; initial set $Y_{i}^{0}$; terminal set $Y_{i}^{\mathrm{end}}$; reliable events $E_{\mathrm{rel}}\subseteq E_{i}$; unreliable events $E_{ur}=E_{i}\setminus E_{\mathrm{rel}}$.**Ensure:** Label $\varphi_{i}:Y_{i}\to \left\{S\mathrm{TRICT},R\mathrm{ECOVERABLE},N\mathrm{ON}\text{-}R\mathrm{OBUST}\right\}$.  1: $B_{\mathrm{rel}}\leftarrow B\mathrm{ACKWARDCLOSURE}\left(Y_{i}^{\mathrm{end}},\;E_{\mathrm{rel}}\right)$▹ states admitting a reliable suffix  2: $R\leftarrow F\mathrm{ORWARD}C\mathrm{LOSURE}R\mathrm{ESTRICTED}(Y_{i}^{0},\;E_{i},\;B_{\mathrm{rel}})$▹ prefix stays inside $B_{\mathrm{rel}}$  3: $S\leftarrow F\mathrm{ORWARD}C\mathrm{LOSURE}R\mathrm{ESTRICTED}(Y_{i}^{0},\;E_{\mathrm{rel}},\;B_{\mathrm{rel}})$▹ reliable prefix inside $B_{\mathrm{rel}}$  4: Rec←R∖S▹ Recoverable but not Strict  5: **for all** 
$y\in Y_{i}$ 
**do**  6:     **if** y∈S  **then**  7:        $\varphi_{i}\left(y\right)\leftarrow \mathrm{Strict}$  8:     **else if** y∈Rec  **then**  9:        $\varphi_{i}\left(y\right)\leftarrow \mathrm{Recoverable}$10:     **else**11:        $\varphi_{i}\left(y\right)\leftarrow \mathrm{Non}\text{-}\mathrm{Robust}$12:     **end if**13: **end for**

The graph primitives invoked by Algorithm 1 are specified in Algorithm 2.

The connection to Algorithm 1 is described below.

Using the localized sets above, we first compute the reliable-suffix basin $B_{\mathrm{rel}}:=$ BackwardClosure$\;\left(Y_{i}^{\mathrm{end}},\;E_{\mathrm{rel}}^{\left(i\right)}\right)$, i.e., states that can reach $Y_{i}^{\mathrm{end}}$ by *reliable* events only. A state has an *arbitrary* (possibly unreliable) prefix whose every prefix remains Recoverable iff it is forward-reachable from $Y_{i}^{0}$ *within* $B_{\mathrm{rel}}$ using labels in $E_{i}$. A state is *Strict* iff, in addition, there exists a *reliable* prefix inside $B_{\mathrm{rel}}$, obtained by forward closure from $Y_{i}^{0}$ with labels in $E_{\mathrm{rel}}^{\left(i\right)}$. Consequently, the classification realizes the logical intent: Strict states admit a reliable prefix and a reliable suffix; Recoverable states admit a path whose every prefix can be recovered by some reliable suffix; and Strict⊆Recoverable holds by construction.
**Algorithm 2** Graph primitives used in Algorithm 1.  1:**function** BackwardClosure(T,A)                        ▹ least fixpoint of X↦T∪Pre(X,A)  2:    R←T  3:     **repeat**  4:        $R\leftarrow T\cup \{\;y\in Y_{i}|\exists a\in A,\;\exists y^{\prime }\in R:\;y\overset{a}{\to }y^{\prime }\;\}$  5:     **until** *R* no longer changes  6:     **return** *R*  7:**end function**  8:**function** ForwardClosureRestricted(S,A,C)                             ▹ BFS restricted to C  9:    Q←S∩C;   R←S∩C10:     **while** Q≠∅  **do**11:        y← pop(Q)12:         **for all** $y\overset{a}{\to }y^{\prime }$ with a∈A  **do**13:            **if** $y^{\prime }\in C$ **and** $y^{\prime }\notin R$  **then**14:               $R\leftarrow R\cup \{y^{\prime }\}$;    push$\left(Q,y^{\prime }\right)$15:            **end if**16:         **end for**17:     **end while**18:     **return** *R*19:**end function**

Complexity: Let $n_{i}:=\left|Y_{i}\right|$ be the number of states of the local automaton $G_{i}$, $m_{i}:=\left|\;\to_{i}\right|$ the number of transitions, and $m_{i}^{\mathrm{rel}}:=\left|\left\{\left(y,a,y^{\prime }\right)\in \to_{i}|a\in E_{\mathrm{rel}}^{\left(i\right)}\right\}\right|$ the number of reliable-labeled transitions. All primitives used by Algorithm 1 are graph traversals (BFS/fixpoints) on finite graphs:

- BackwardClosure$\left(Y_{i}^{\mathrm{end}},\;E_{\mathrm{rel}}^{\left(i\right)}\right)$: reverse-BFS/least-fixpoint over reliable edges; it runs in $O(n_{i}+m_{i}^{\mathrm{rel}})$ time and $O(n_{i})$ memory.
- ForwardClosureRestricted$\left(Y_{i}^{0},\;E_{i},\;B_{\mathrm{rel}}\right)$: BFS restricted to $B_{\mathrm{rel}}$; it runs in $O(n_{i}+m_{i})$ time and $O(n_{i})$ memory.
- ForwardClosureRestricted$\left(Y_{i}^{0},\;E_{\mathrm{rel}}^{\left(i\right)},\;B_{\mathrm{rel}}\right)$: BFS on the reliable subgraph inside $B_{\mathrm{rel}}$; it runs in $O(n_{i}+m_{i}^{\mathrm{rel}})$ time and $O(n_{i})$ memory.
- The final labeling loop over $Y_{i}$ is $O(n_{i})$.

Hence, Algorithm 1 runs in overall time

$$O\;\left(n_{i}+m_{i}\right)\;\mathrm{and}\;\mathrm{uses}\;O\left(n_{i}\right)\;\mathrm{memory},$$

i.e., linear in the size of the local graph. We compute all detector scales as $\sum_{i=1}^{N}O\left(n_{i}+m_{i}\right)$.

To enhance the interpretability of robustness classification under local observations, we visualize the reachable state space of the local detector $D_{i}$ for subsystem $G_{1}$.

To further explain the interpretation of each detector state in subsystem $G_{1}$, we list below the component-wise markings in Table 1 of $y_{i}^{\left(1\right)}\in Y_{D_{1}}$, which represent the synchronized configurations of product and resource automata.

The same modeling principle and robustness classification procedure are applied to the remaining subsystems $G_{2}$–$G_{4}$, but their detector state table is omitted for brevity.

To intuitively present the results of the local robust state classification, we provide the robustness-annotated state transition diagram for subsystem $G_{1}=G_{P_{1}}\left\|G_{R_{1}}\right\|G_{R_{2}}$. Each state $y_{i}\in Y_{D_{i}}$ is colored according to its robustness label.

**Figure 6:**
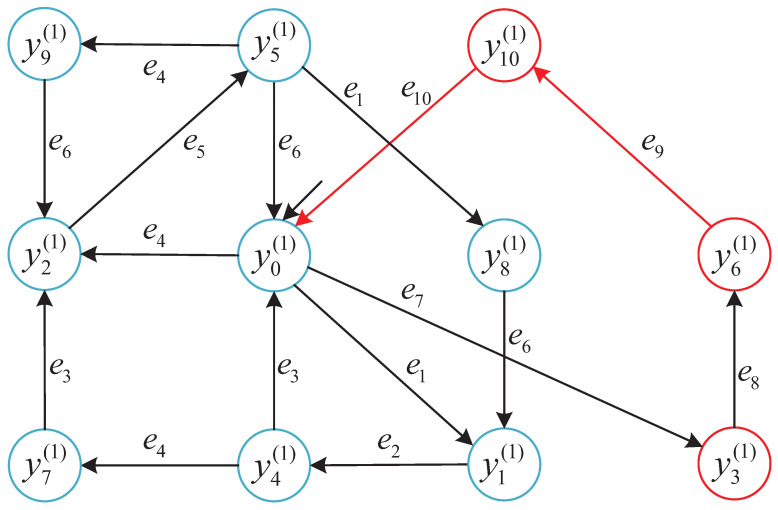
Local state detector of subsystem $G_{1}$ with robustness labels. Blue nodes: Strict; red nodes: Non-robust. (There are no Recoverable-only (green) states in this subgraph.) Red arrows: $E_{\mathrm{ur}}$; black arrows: $E_{\mathrm{rel}}$.

The states in Figure 6 correspond to $Y_{D_{i}}=\left\{y_{0},y_{1},...,y_{10}\right\}$.

**Figure 7:**
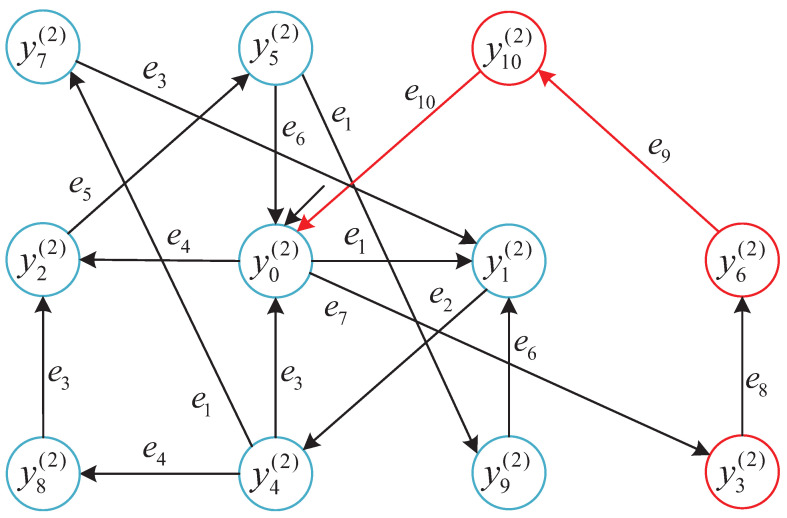
Robustness-classified local state detector $D_{i}$ for subsystem $G_{2}$. Blue states are Strictly (and Recoverably) robust; red states are Non-robust. Red edges indicate unreliable events ($E_{ur}$); black edges indicate reliable events.

**Figure 8:**
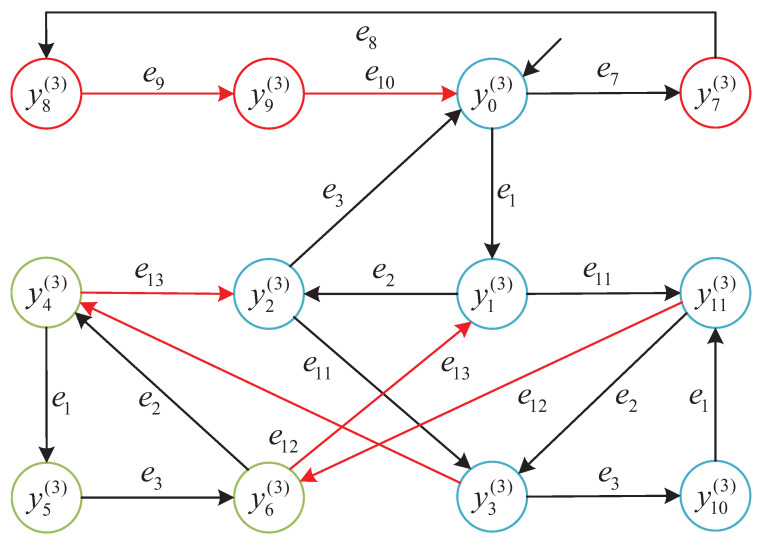
Partial detector of $G_{3}$ showing representative robustness-relevant paths. Blue nodes: Strict; green nodes: Recoverable-only (Rec∖Strict); red nodes: Non-robust. Labels are computed per state by Algorithm 1 and are path-independent. Red arrows: $E_{\mathrm{ur}}$; black arrows: $E_{\mathrm{rel}}$.

**Figure 9:**
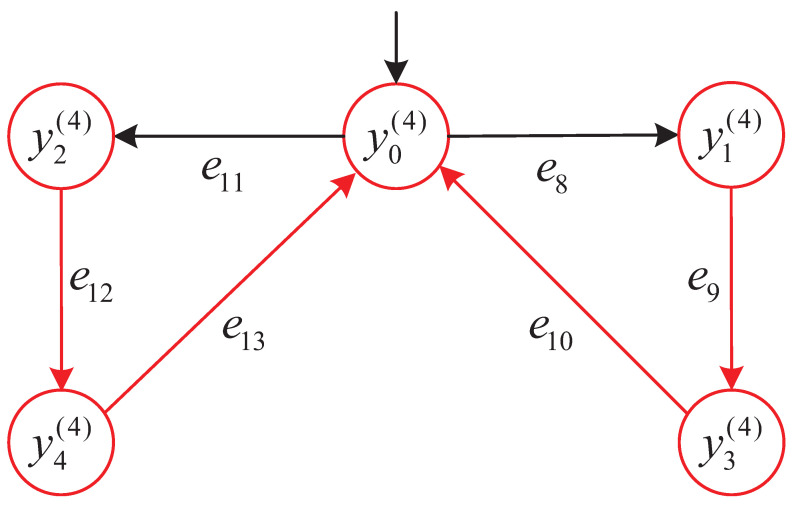
All states in this diagram are Non-robust due to the presence of unrecoverable transitions triggered by unreliable events. Red edges: unreliable; black edges: reliable.

This structure reveals how unreliable events (e.g., $e_{9}$, $e_{10}$) impact recoverability. The same method is applied to other subsystems in the following Figure 7, Figure 8 and Figure 9.

To improve clarity, we extract a representative fragment of the detector $D_{3}$ in subsystem $G_{3}$, emphasizing robustness-relevant states and transitions. Unlike previous subsystems, $G_{3}$ contains intermediate states whose robustness classification depends on the presence of unreliable events in the prefix path.

Unlike the detectors for $G_{1}$ and $G_{2}$, the robustness structure of $G_{4}$ is dominated by Non-robust states. This is due to the fact that many transitions in $G_{4}$ involve unreliable events, forming unrecoverable cycles or branches. Thus, local supervisory control in this subsystem must Strictly avoid enabling transitions such as $e_{9}$, $e_{10}$, $e_{12}$, and $e_{13}$.

### 4.2. Global Robustness Classification and Guarantee

To enable distributed robustness enforcement, each local supervisor $S_{i}$ must make control decisions based on its current detector state $y_{i}$. The robustness classification $\varphi_{i}\left(y_{i}\right)$ determines which controllable events are allowed, depending on whether the state is Strict, Recoverable, or Non-robust.

Notation. For $e\in E_{i}$ and $y_{i}\in Y_{i}$, define $Post_{e}\left(y_{i}\right)≜\left\{\;y_{i}^{\prime }\in Y_{i}|y_{i}\overset{e}{\to }y_{i}^{\prime }\;\right\}$. A shared event is globally enabled at a joint state $y={\left(y_{i}\right)}_{i\in N}$ iff it is enabled by every local supervisor that synchronizes on it (conjunctive fusion).

Below, Algorithm 3 summarizes the local decision-making rule.
**Algorithm 3** Local supervisor enabling rule based on robustness.**Require:** Local observation $s_{o}\in {\left(E_{i}^{0}\right)}^{*}$**Ensure:** Enabled set $S_{i}\left(s_{o}\right)\subseteq E_{i}$    *(here $Strict_{i}$ and $Rec_{i}$ are the offline partitions precomputed by Algorithm 1).*1: $y_{i}\leftarrow f_{D_{i}}\left(y_{i0},s_{o}\right)$▹ current detector state2: $r\leftarrow \varphi_{i}\left(y_{i}\right)$▹ robustness label3: **if** *r* = Strict **then**4:    $S_{i}\left(s_{o}\right)\leftarrow \left\{\;e\in E_{i}|Post_{e}y_{i}\subseteq {\mathrm{Strict}}_{i}\;\right\}$5: **else if** *r* = Recoverable **then**6:    $S_{i}\left(s_{o}\right)\leftarrow \left\{\;e\in E_{i}|Post_{e}y_{i}\subseteq {\mathrm{Rec}}_{i}\;\right\}$7: **else**8:    $S_{i}\left(s_{o}\right)\leftarrow ⌀$9: **end if**

Label order and global aggregation:

Let the label set be L={Non-robust,Recoverable,Strict} endowed with the total order Non-robust⪯Recoverable⪯Strict. For a joint detector state $y={\left(y_{i}\right)}_{i\in N}$, the global robustness label is

$$\varphi_{\mathrm{global}}\left(y\right)\;=\;\min_{⪯,\;i\in N}\;\varphi_{i}\left(y_{i}\right),$$

where $\min_{⪯}$ denotes the minimum with respect to the above total order (i.e., the weakest label dominates). Equivalently,

$$\varphi_{\mathrm{global}}\left(y\right)\;=\;\left\{\begin{array}{cc} \mathrm{Strict},\; & \mathrm{if}\;\forall i:\;\varphi_{i}\left(y_{i}\right)=\mathrm{Strict},\\ \mathrm{Recoverable},\; & \mathrm{if}\;\left(\forall i:\;\varphi_{i}\left(y_{i}\right)\in \left\{\mathrm{Strict},\mathrm{Rec}\right\}\right)\;\wedge \;\left(\exists j:\;\varphi_{j}\left(y_{j}\right)=\mathrm{Rec}\right),\\ \mathrm{Non}\text{-}\mathrm{robust},\; & \mathrm{otherwise}. \end{array}\right.$$

**Lemma** **2.** *(Global robustness consistency.) If any local detector $D_{i}$ classifies a state as Non-robust, then the global system state is Non-robust.*

**Proof.**  By definition, global robustness classification is determined by the least robust local detector classification. Hence, a Non-robust local classification directly yields a global Non-robust state.    □

**Lemma** **3.** *(Robustness propagation.) If all local detectors $D_{i}$ classify their current state $y_{i}$ as either Strict or Recoverable, then the global state is at least Recoverable.*

**Proof.**  By definition of the global classification rule $\varphi_{global}\left(y\right)=\min_{⪯,\;i\in N}\varphi_{i}\left(y_{i}\right)$, where $\varphi_{i}\in \left\{Strict,Recoverable,Non\text{-}robust\right\}$, the absence of any Non-robust local state ensures that $\varphi_{global}\left(y\right)\in \left\{Strict,Recoverable\right\}$.    □

**Theorem** **1** (Global robustness under reliable-event enforcement)**.** *Let ${G=\|}_{i\in N}G_{i}$ be the synchronous product of local automata. For each i, Algorithm 1 computes a partition $Y_{i}=Strict_{i}\;⊎\;\left(Rec_{i}\setminus Strict_{i}\right)\;⊎\;Non\text{-}robust_{i}$ with $Strict_{i}\subseteq Rec_{i}$, where $Strict_{i}$ (resp. $Rec_{i}$) is the set of states that admit a reliable prefix (resp. a prefix whose every prefix remains Recoverable by some reliable suffix) to the terminal set $Y_{i}^{\mathrm{end}}$.*

*Each local supervisor $S_{i}$ applies the following reliable-state rule at its current detector state $y_{i}$:*

$$\left\{\begin{array}{cc} y_{i}\in Strict_{i}: & \mathrm{enable}\;\mathrm{exactly}\;\mathrm{the}\;\mathrm{events}\;e\in E_{i}\;\mathrm{with}\;Post_{e}\left(y_{i}\right)\subseteq Strict_{i},\\ y_{i}\in Rec_{i}\setminus Strict_{i}: & \mathrm{enable}\;\mathrm{exactly}\;\mathrm{the}\;\mathrm{events}\;e\in E_{i}\;\mathrm{with}\;Post_{e}\left(y_{i}\right)\subseteq Rec_{i},\\ y_{i}\in Non\text{-}robust_{i}: & \mathrm{disable}\;\mathrm{all}\;\mathrm{events}\;\mathrm{in}\;E_{i}. \end{array}\right.$$

*A shared event is globally enabled at a global state $y={\left(y_{i}\right)}_{i\in N}$ iff it is enabled by* every *local supervisor that synchronizes on it (conjunctive fusion).*

*Then along every closed-loop execution, each visited global state y satisfies $y_{i}\in Rec_{i}$ for all i∈N. Equivalently, the global robustness label*

$$\varphi_{\mathrm{global}}\left(y\right)\;:=\;\min_{⪯,\;i\in N}\;\varphi_{i}\left(y_{i}\right)\;\left(Strict≻Recoverable≻Non\text{-}robust\right)$$

*always belongs to {Strict,Recoverable}. Moreover,*

$$\begin{array}{cc} \; & \varphi_{\mathrm{global}}\left(y\right)=Strict\Leftrightarrow \forall i\in N:\;y_{i}\in Strict_{i},\\ \; & \varphi_{\mathrm{global}}\left(y\right)=Recoverable\Leftrightarrow \left(\exists i:\;y_{i}\in Rec_{i}\setminus Strict_{i}\right)\;\wedge \;\left(\forall i:\;y_{i}\notin Non\text{-}robust_{i}\right). \end{array}$$

**Proof.**  We show that $\prod_{i\in N}Rec_{i}$ is an invariant of the closed loop.

By Algorithm 1, we first compute the reliable-suffix basin $B_{\mathrm{rel}}=\mathrm{BackwardClosure}\left(Y_{i}^{\mathrm{end}},E_{\mathrm{rel}}^{\left(i\right)}\right)$ and then $R=\mathrm{ForwardClosureRestricted}(Y_{i}^{0},E_{i},B_{\mathrm{rel}})$; hence, $Rec_{i}=R\setminus Strict_{i}$ collects exactly the states that are forward-reachable from $Y_{i}^{0}$ while staying inside $B_{\mathrm{rel}}$. In particular, $y_{i}^{0}\in Rec_{i}$, so the initial global state $y^{0}={\left(y_{i}^{0}\right)}_{i\in N}$ belongs to $\prod_{i}Rec_{i}$.

Let $y={\left(y_{i}\right)}_{i\in N}\in \prod_{i}Rec_{i}$ and suppose a global event *e* occurs to $y^{\prime }={\left(y_{i}^{\prime }\right)}_{i\in N}$. Because all events are controllable and the global enabling is conjunctive, *e* can occur only if each involved supervisor $S_{i}$ enables *e* at $y_{i}$. If $y_{i}\in Strict_{i}$, the rule enables only transitions with $Post_{e}\left(y_{i}\right)\subseteq Strict_{i}$, where $Post_{e}\left(y_{i}\right)≜\left\{y_{i}^{\prime }|y_{i}\overset{e}{\to }y_{i}^{\prime }\right\}$, thus $y_{i}^{\prime }\in Strict_{i}\subseteq Rec_{i}$. If $y_{i}\in Rec_{i}\setminus Strict_{i}$, the rule enables only transitions with $Post_{e}\left(y_{i}\right)\subseteq Rec_{i}$, hence $y_{i}^{\prime }\in Rec_{i}$. If for some *i* we had $y_{i}\in Non\text{-}robust_{i}$, no event would be enabled contradicting the occurrence of *e* at *y*. Therefore, $y^{\prime }\in \prod_{i}Rec_{i}$ and the invariant holds.

Consequently, no closed-loop execution can reach a local Non-robust state, i.e., $\varphi_{i}\left(y_{i}\right)\in \left\{Strict,Recoverable\right\}$ for all *i*, so $\varphi_{\mathrm{global}}\left(y\right)=\min_{i}\varphi_{i}\left(y_{i}\right)\in \left\{Strict,Recoverable\right\}$. The two characterizations of $\varphi_{\mathrm{global}}$ follow directly from the order Strict≻Recoverable≻Non-robust and the invariance $\prod_{i}Rec_{i}$.    □

We construct a robust event enablement, see Table 2, mapping local detector states to enabled events.

Table 2, which shows robust events, assists the local supervisors in making rapid and precise decisions.

The next chapter provides experimental validation of the distributed robust supervisory strategy, verifying its efficacy under practical scenarios.

### 4.3. Structural Characterization of Local Robustness

The robustness of each subsystem $G_{i}$ is structurally influenced by its topological configuration and its interaction with unreliable events. In particular, the existence of cycles or interleaving paths involving events in $E_{ur}$ directly affects the classification of states in $Y_{D_{i}}$.

We formally observe the following:

**Lemma** **4** (Structural strictness via reliable closures)**.** *For subsystem $G_{i}$ with reliable alphabet $E_{\mathrm{rel}}^{\left(i\right)}$, define*

$$F_{\mathrm{rel}}≜ForwardClosure\left(Y_{i}^{0},\;E_{\mathrm{rel}}^{\left(i\right)}\right),\;B_{\mathrm{rel}}≜BackwardClosure\left(Y_{i}^{\mathrm{end}},\;E_{\mathrm{rel}}^{\left(i\right)}\right).$$

*Then a state $y\in Y_{D_{i}}$ is* Strictly robust *iff $y\in F_{\mathrm{rel}}\cap B_{\mathrm{rel}}$.*

**Lemma** **5** (Cycle-induced Non-robustness (sufficient))**.** *If a state y lies on a cycle that contains some event in $E_{\mathrm{ur}}^{\left(i\right)}$ and $y\notin B_{\mathrm{rel}}$, then y is classified as Non-robust.*

**Figure 10:**
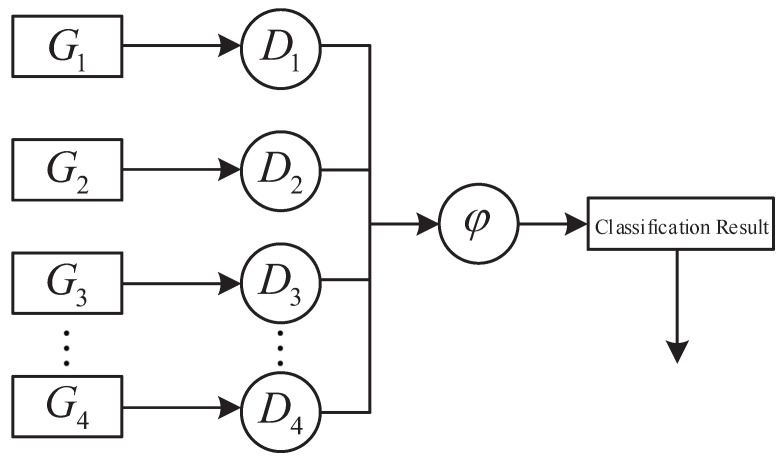
Distributed robust control structure with local detectors and global merging via φ rule. The classification result determines the global robustness label of the current joint state, guiding the distributed execution decision.

To summarize the overall distributed control flow, Figure 10 illustrates the high-level architecture of the proposed framework, where each subsystem constructs a local detector, and the global classification is obtained through the merging rule φ.

## 5. Experimental Validation

To evaluate the applicability of the proposed framework in automated and micro-manufacturing contexts, simulation studies are conducted on representative system models. All subsystem automata and detector structures were constructed based on the modular modeling approach proposed in Section 3. The robustness classification Algorithms 1 and 3 were implemented using a Java-based simulation framework developed by the authors. All simulations were executed on a Windows 10 workstation with an Intel Core i7 processor and 16 GB RAM.

To visualize the local detectors and robustness propagation structures, we manually generated state transition graphs using Microsoft Visio. Each diagram reflects the formal construction of detectors based on synchronous composition and robustness labeling, as defined in Section 4.

### 5.1. Experimental Setup

Each local plant $G_{i}$ is modeled as the synchronous composition of a product automaton $G_{P_{i}}$ and its corresponding resource automata $\left\{G_{R_{j}}\right\}$:

$$G_{i}=G_{P_{i}}\|\left({\|}_{j\in J_{i}}G_{R_{j}}\right),$$

where $J_{i}$ denotes the index set of resources used by subsystem *i*. Four distributed subsystems are constructed as follows:

- $G_{1}=G_{P_{1}}\left\|G_{R_{1}}\right\|G_{R_{2}}$;
- $G_{2}=G_{P_{2}}\left\|G_{R_{1}}\right\|G_{R_{2}}$;
- $G_{3}=G_{P_{3}}\|G_{R_{1}}\|G_{R_{2}}\left\|G_{R_{3}}\right\|G_{R_{4}}$;
- $G_{4}=G_{P_{4}}\left\|G_{R_{3}}\right\|G_{R_{4}}$.

Unreliable events are defined as $E_{ur}=\left\{e_{9},e_{10},e_{12},e_{13}\right\}$, corresponding to typical faults:

- $e_{9}$: tool jam or axis over-current on a precision stage;
- $e_{10}$: part/wafers misalignment detected by the vision system;
- $e_{12}$: loss of vacuum or gripping failure during pick-place;
- $e_{13}$: vision reject after micro-inspection.

A compact mapping is summarized in Table 3. These faults occur at the event granularity and directly impact shared resources, which is consistent with our robustness classification and local supervision.

### 5.2. Robustness Structure Analysis of Subsystems

We summarize the robustness classification across subsystems based on the constructed detector automata, as visualized in Figure 6, Figure 7, Figure 8 and Figure 9. Each figure highlights state robustness categories under local observation:

- **$G_{1}$** and **$G_{2}$** contain mostly Strictly robust states, with only a few Non-robust configurations.
- **$G_{3}$** exhibits a mixed structure, including ambiguous and Recoverable states due to its complex resource interactions.
- **$G_{4}$** is entirely Non-robust, as every reachable state involves unreliable transitions.

These results validate the effectiveness of local detectors in classifying robustness-critical regions, which directly influence supervisory control strategies. A summary of robustness distribution is given in Table 4.

### 5.3. Supervisor Response to Event Sequences

Each local supervisor $S_{i}$ applies the robust event-enablement rule using its detector $D_{i}$ to classify states and selectively disable risky transitions.

In $G_{1}$ and $G_{2}$, only Strictly robust paths are allowed. In $G_{3}$, the supervisor permits Recoverable trajectories while blocking transitions to Non-robust regions. In $G_{4}$, all controllable transitions are disabled to avoid Non-robust cycles.

### 5.4. Structural Sensitivity and Scalability

Robustness is structurally influenced by the interaction between subsystems and unreliable events. As interleaving increases, detectors grow in size and more states become ambiguous or Non-robust. In $G_{3}$, complex resource sharing induces a significant number of states requiring Recoverable supervision. In contrast, $G_{4}$ demonstrates the fragility of subsystems lacking redundant safe paths.

### 5.5. Robustness Enforcement Consistency

The consistency between local supervisory decisions and global robustness outcomes is confirmed through representative simulations. The following is shown in Figure 6, Figure 7, Figure 8 and Figure 9 and Section 5.2:

- Supervisors in $G_{1}$ and $G_{2}$ enable only *Strictly robust* trajectories, which are also a subset of Recoverably robust behaviors.
- Supervisors in $G_{3}$ permit *Recoverable* but not Strictly robust trajectories while preventing unsafe transitions.
- Supervisors in $G_{4}$ disable all controllable events due to inherent Non-robustness.

These results confirm that the distributed supervisory strategy achieves global robustness through local enforcement without requiring centralized coordination.

### 5.6. Evaluation of Structural Performance Metrics

To complement the robustness enforcement results presented in Section 5.2, Section 5.3, Section 5.4 and Section 5.5, this section focuses on architectural-level performance indicators that reflect the practical value of the proposed distributed framework. Instead of measuring time-based performance—often dependent on platform-specific implementation—we evaluate structural properties that are stable across platforms and scale with system complexity. Specifically, we compare centralized and distributed designs in terms of reachable states, modularity, and extensibility.

We consider a partial plant composed of subsystems $G_{1}$ and $G_{2}$, defined as

$$\begin{array}{cc} G_{1} & =G_{P_{1}}\left\|G_{R_{1}}\right\|G_{R_{2}},\\ G_{2} & =G_{P_{2}}\left\|G_{R_{1}}\right\|G_{R_{2}}. \end{array}$$

The centralized plant is constructed as

$$G_{c}=G_{P_{1}}\|G_{P_{2}}\left\|G_{R_{1}}\right\|G_{R_{2}},$$

while the distributed approach constructs independent local detectors $D_{1}$ and $D_{2}$ over $G_{1}$ and $G_{2}$, respectively. These subsystems and their corresponding structures are consistent with the plant models illustrated in Figure 2 and Figure 3 and the detector graphs in Figure 6, Figure 7, Figure 8 and Figure 9.

Table 5 summarizes the structural performance differences between the centralized and distributed architectures in terms of reachability, design reusability, and scalability.

Note: ‘Reachable States’ counts the reachable states of the detector(s)—a single monolithic detector for the centralized case or the sum of local detectors for the distributed case—not the size of the plant’s global synchronous product.

Scalability: The complexity of each local detector $D_{i}$ scales with the size of its local graph only. Algorithm 1 runs in $O(|Y_{i}|+|\to_{i}|)$ time and uses $O(|Y_{i}|)$ memory, where $Y_{i}$ and $\to_{i}$ are the reachable states and edges of the detector built on the local alphabet $E_{i}$ (product automaton of $G_{P_{i}}$ with its adjacent resources). Thus, growth is driven by the local interaction degree $d_{i}$ (number of shared resources/events), not by the total number of subsystems *N*. In our case study, the most connected subsystem $G_{3}$ yields >30 detector states, while $G_{1}$ and $G_{2}$ each have 11, showing that ‘hub’ modules can dominate the footprint, whereas other modules remain small. Practical mitigations include resource partitioning/decoupling, clustering or hierarchical detectors for hubs, and event abstraction to shrink $E_{i}$. Here are some examples:

- The centralized model has fewer reachable states due to full synchronization and global pruning.
- The distributed approach constructs reusable local detectors that enable modular expansion.
- Adding new modules (e.g., $G_{3}$, $G_{4}$) in the centralized case requires reconstructing the full plant, while distributed controllers support incremental composition.

These metrics collectively confirm that although centralized synthesis produces compact models for small configurations, the distributed approach offers substantial structural advantages. It supports subsystem-level reuse, incremental integration, and scalable extension to larger system configurations—all without requiring full plant redesign.

### 5.7. Language-Level Comparison of Centralized and Distributed Control

To complement the structural evaluation presented in Section 5.6, we now compare the behavioral correctness of the proposed distributed supervisory scheme against a centralized baseline. The objective is to verify whether the distributed controllers can achieve equivalent robustness enforcement in terms of permissible event sequences and rejection of unsafe trajectories.

We consider the same partial plant $G=G_{1}\|G_{2}$ and its centralized composition $G_{c}=G_{P_{1}}\|G_{P_{2}}\left\|G_{R_{1}}\right\|G_{R_{2}}$, as defined in Section 5.6. The centralized supervisor is synthesized over $G_{c}$ using robustness enforcement based on Recoverably robust trajectories. The distributed control scheme uses two detectors $D_{1}$ and $D_{2}$ to determine local robustness and enable event decisions based on the distributed rule:

$$S\left(s\right)=\overset{2}{\underset{i=1}{⋂}}S_{i}\left(P_{i}\left(s\right)\right),$$

where $P_{i}\left(s\right)$ denotes the projection of string *s* onto the event set of $G_{i}$.

Let $L_{c}$ denote the language generated by the centralized supervisor and $L_{d}$ denote the language generated by the distributed scheme under detector-based coordination.

We now state a formal result that characterizes the behavioral soundness of the distributed supervisor.

**Proposition** **2.** *Let $G=G_{1}\|G_{2}$ and let $D_{1}$, $D_{2}$ be the robust state detectors constructed for $G_{1}$ and $G_{2}$, respectively. Let $L_{c}$ be the language of the centralized robust supervisor synthesized over $G_{c}=G_{P_{1}}\|G_{P_{2}}\left\|G_{R_{1}}\right\|G_{R_{2}}$, and let $L_{d}=\left\{s\in L\left(G\right)|D_{i}\left(P_{i}\left(s\right)\right)\neq \emptyset \;for\;all\;i=1,2\right\}$ be the language permitted by the distributed strategy.*

*Then, the following holds:*

*1*. *$L_{d}\subseteq L_{c}$;*
*2*. *$L\left(G\right)\setminus L_{d}\subseteq L\left(G\right)\setminus L_{c}$.*

**Proof.**  Consider any string $s\in L_{d}$. By definition of the distributed strategy, $D_{1}\left(P_{1}\left(s\right)\right)\neq \emptyset$ and $D_{2}\left(P_{2}\left(s\right)\right)\neq \emptyset$. According to the detector design (see Algorithm 1), this means that $P_{1}\left(s\right)$ leads to a state in $Y_{D}^{\left(1\right)}$ and $P_{2}\left(s\right)$ leads to a state in $Y_{D}^{\left(2\right)}$, both of which are either Strictly robust or Recoverably robust.

Since the centralized supervisor $L_{c}$ is synthesized over $G_{c}$, which contains the full behavior of *G*, and robustness pruning only removes strings violating Definition 3, any *s* that passes all local robustness detectors also satisfies the global robustness condition. Thus, $s\in L_{c}$ and $L_{d}\subseteq L_{c}$.

Now consider any string $s\in L\left(G\right)\setminus L_{d}$. Then there exists some *i* such that $D_{i}\left(P_{i}\left(s\right)\right)=\emptyset$, meaning $P_{i}\left(s\right)$ leads to a Non-robust state in $G_{i}$. Since the global supervisor enforces robustness over all components, such a string must also be removed from $L_{c}$ during centralized pruning. Therefore, $s\notin L_{c}$ and $s\in L\left(G\right)\setminus L_{c}$, implying $L\left(G\right)\setminus L_{d}\subseteq L\left(G\right)\setminus L_{c}$.    □

### 5.8. Trajectory-Level Validation of Recoverable Robustness

While the structural and language-level analyses in Section 5.6 and Section 5.7 demonstrate that the distributed control framework enforces robustness consistently, it remains essential to validate that the detectors allow appropriate runtime execution for strings that meet the Recoverable robustness condition. In this section, we evaluate a specific trajectory under the distributed supervisory scheme to illustrate the runtime behavior and semantic interpretation of Recoverable robustness.

We consider the subsystem $G_{3}$ with its detector $D_{3}$ illustrated in Figure 8. The following event sequence is analyzed:

$$s=e_{1}e_{2}e_{11}e_{12}e_{1}e_{3}.$$

This path contains the unreliable event $e_{12}\in E_{ur}$ and tests the distributed supervisor’s ability to accept partially unreliable behavior while preserving the ability to reach a terminal configuration.

Let *y* denote the state reached after executing the prefix $e_{1}e_{2}e_{11}e_{12}$. According to the detector classification in $D_{3}$, the state $y=y_{4}^{\left(3\right)}$ is labeled as Recoverably robust. This classification is justified because, although $e_{12}$ is an unreliable event, there exists a valid recovery sequence $e_{1}e_{3}$ from *y* that leads to the terminal state $Y^{\mathrm{end}}$. Thus, the Recoverably robust label permits continuation beyond an unreliable event provided a complete recovery path is guaranteed.

At the detector state $y=y_{4}^{\left(3\right)}$, the supervisor evaluates whether to allow the event $e_{1}$ to continue execution. Since the detector classification is Recoverable and a valid recovery trajectory exists via $e_{3}$, the system permits $e_{1}$ to occur.

After executing $e_{1}$ and then $e_{3}$, the system reaches the terminal configuration $y_{6}^{\left(3\right)}$. According to the terminal state definition introduced in Section 2.4, $y_{6}^{\left(3\right)}$ lies in the local terminal state set $Y_{\left(3\right)}^{\mathrm{end}}$ of subsystem $G_{3}$. This demonstrates that the recovery path exists and is operational, validating the runtime semantics of the Recoverably robust label and confirming that the execution leads to a semantically complete production cycle.

**Figure 11:**
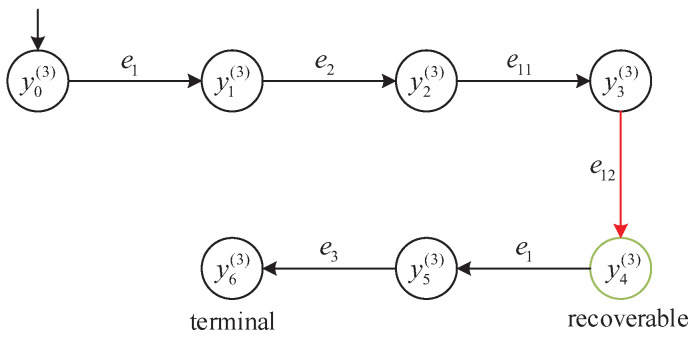
Trajectory segment illustrating Recoverable-robustness enforcement along the path $s=e_{1}e_{2}e_{11}e_{12}e_{1}e_{3}$. Red arc $e_{12}$: unreliable event $\left(e_{12}\in E_{ur}\right)$. Black arcs: reliable events. Green-highlighted state $y_{4}^{\left(3\right)}$: Recoverable (a recovery sequence $e_{1}e_{3}$ leads to the terminal state $y_{6}^{\left(3\right)}$.

Figure 11 visualizes the state transitions along the trajectory *s*, highlighting the transitions involving unreliable events and the eventual recovery via $e_{3}$. The states along the path are as follows:

- $y_{0}^{\left(3\right)}$(initial)$\overset{e_{1}}{\to }$$y_{1}^{\left(3\right)}$;
- $y_{1}^{\left(3\right)}\overset{e_{2}}{\to }y_{2}^{\left(3\right)}$;
- $y_{2}^{\left(3\right)}\overset{e_{11}}{\to }y_{3}^{\left(3\right)}$;
- $y_{3}^{\left(3\right)}\overset{e_{12}}{\to }y_{4}^{\left(3\right)}$(Recoverable robustness);
- $y_{4}^{\left(3\right)}\overset{e_{1}}{\to }y_{5}^{\left(3\right)}\overset{e_{3}}{\to }y_{6}^{\left(3\right)}$(terminal).

This example confirms that the distributed supervisor correctly interprets the Recoverably robust classification and allows execution of unreliable events only when a valid recovery path exists. Such behavior illustrates the semantic soundness of the detector-based control strategy in runtime decision-making.

These structural properties confirm the feasibility of implementing the proposed detector-based supervisors in real-world industrial environments. In particular, the modular architecture is well-suited for deployment on edge controllers or Programmable Logic Controllers (PLCs) within smart factories, enabling real-time detection and mitigation of failures without centralized coordination.

### 5.9. Comparison with Existing Methods

To further assess the efficacy of the proposed distributed framework, we qualitatively compare it with several representative supervisory-control approaches: centralized robust synthesis, static fault-tolerant control (FTC), modular supervision, and our distributed robust scheme (see Table 6). The comparison focuses on unreliable-event handling, Recoverable robustness, state-space scalability, supervisor reusability, and incremental integration.

The centralized strategy provides strong guarantees but suffers from state explosion and poor scalability. Static FTC approaches enable some resilience, yet they typically rely on predefined failure models and cannot adapt at runtime. Modular architectures support structural reuse but lack explicit robustness enforcement against event uncertainty.

Compared with a centralized robust supervisor, the proposed distributed scheme achieves the same safety guarantees (see Proposition 2 and Section 5.7) while avoiding global runtime search. At runtime, decisions are made locally by inspecting the label of the current detector state and a small set of enabled events; therefore, the decision cost depends on the local event alphabet and the detector size rather than the size of the monolithic product automaton (Table 6). In practice, this reduces decision latency and improves controller reuse and scalability (cf. Section 5.6 and Table 5). Because timing strongly depends on platform and implementation (PLC vs. ROS, CPU load, I/O latency, etc.), we deliberately do not report a fixed percentage improvement and leave a cross-platform timing benchmark as future work.

The detector-based control structure is amenable to modular implementation, since each local subsystem requires only partial event monitoring and local classification. The architecture is compatible with PLC-based or ROS-based deployments and supports incremental system expansion and fault isolation, and it is therefore a strong candidate for practical cyber–physical manufacturing environments.

Practical implementation for micro-manufacturing: Each detector can be deployed as local logic on PLCs (IEC 61131-3 [31]) or ROS edge controllers. Because only partial events are monitored and the detector automaton is small, runtime checks are constant-time with negligible memory footprint compared to a monolithic product automaton. This enables short-cycle reactions, fault isolation at the cell level, and incremental expansion of production lines—key properties for micro-manufacturing and MEMS-based execution platforms (see also Section 5.1/Table 3).

Network latency and deployment: Our enabling rule is conjunctive and event-driven without a global clock. Latency mainly affects throughput (waiting for all involved local supervisors to enable a shared event) but not the correctness of the robustness guarantee. For time-critical shared operations, we recommend co-locating the relevant supervisors with the shared resource (e.g., on the same PLC rack or ROS edge) or using industrial fieldbuses with bounded jitter. A quantitative latency budget and its impact on cycle time are part of our planned hardware testbed.

### 5.10. Cost and Scalability Micro-Study

Setup: We use the running example with N=4 product automata and 4 resource automata. For each local composition $G_{i}=\left(Y_{i},E_{i},\to_{i}\right)$, we run Algorithm 1 once and record the following platform-agnostic counters: (i) graph sizes $n_{i}\;=\;\left|Y_{i}\right|$, $m_{i}\;=\;\left|\to_{i}\right|$, and $m_{i}^{\mathrm{rel}}\;=\;\left|\left\{\left(y,a,y^{\prime }\right)\;\in \;\to_{i}|a\;\in \;E_{\mathrm{rel}}^{\left(i\right)}\right\}\right|$; (ii) the intermediate sets of Algorithm 1: $|B_{\mathrm{rel}}|$, |R|, |S|; and (iii) the final label counts $|{\mathrm{Strict}}_{i}|$, $|{\mathrm{Rec}}_{i}|$, $|Non\text{-}robust_{i}|$.

Unless otherwise stated, the terminal set $Y_{i}^{\mathrm{end}}$ used by Algorithm 1 is obtained by the product–events semantics: let $E_{\mathrm{term}}^{\mathrm{prod}}$ be the union of terminal events declared by the product automata. Whenever a shared event $a\;\in \;E_{\mathrm{term}}^{\mathrm{prod}}$ fires in the synchronous product that defines $G_{i}$, the successor global state is inserted into $Y_{i}^{\mathrm{end}}$. Multiple successors reached by the same terminal event at the *same* global state are *deduplicated*. Reliable events are $E_{\mathrm{rel}}^{\left(i\right)}\;=\;E_{i}\setminus E_{\mathrm{ur}}^{\left(i\right)}$ as in Section 4, and all events are controllable/observable (Assumption 1 in Section 3).

Centralized structural baseline: As a baseline, we build once the synchronous product of the eight base automata are reached and report the reachable sizes |X| and |⇒|. Table 7 compares the centralized reachable graph with the sum of locals. We also report the (purely structural) explosion factors

$${\mathrm{EF}}_{\mathrm{states}}=\frac{\left|X\right|}{\sum_{i}n_{i}},\;{\mathrm{EF}}_{\mathrm{edges}}=\frac{\left|\Rightarrow \right|}{\sum_{i}m_{i}}.$$

These ratios quantify how much larger the centralized model is than the aggregate of local models, independent of execution platforms.

Workload of Algorithm 1 (platform-agnostic): For each $G_{i}$ we also accumulate the edge visits of the three graph primitives used by Algorithm 1 (Algorithm 2):

- $c_{i}^{\mathrm{back}}$: number of reliable edges inspected by BackwardClosure$\left(Y_{i}^{\mathrm{end}},E_{\mathrm{rel}}^{\left(i\right)}\right)$;
- $c_{i}^{\mathrm{all}}$: number of edges scanned by ForwardClosureRestricted$\left(Y_{i}^{0},E_{i},B_{\mathrm{rel}}\right)$;
- $c_{i}^{\mathrm{rel}}$: number of reliable edges scanned by ForwardClosureRestricted$\left(Y_{i}^{0},E_{\mathrm{rel}}^{\left(i\right)},B_{\mathrm{rel}}\right)$.

And we also accumulate the peak queue length $q_{i}^{\max}$ among these BFS/fixpoint procedures, which serves as a proxy for memory usage. Table 8 summarizes the counts. In all cases, the total work $c_{i}^{\mathrm{back}}+c_{i}^{\mathrm{all}}+c_{i}^{\mathrm{rel}}$ empirically matches the linear-time bound $O(n_{i}+m_{i})$ on $G_{i}$.

Label distributions (Strict ⊆ Rec): To avoid ambiguity and to respect ${\mathrm{Strict}}_{i}\subseteq {\mathrm{Rec}}_{i}$, we report $|{\mathrm{Strict}}_{i}|$, $|{\mathrm{Rec}}_{i}\setminus {\mathrm{Strict}}_{i}|$, their sum $|{\mathrm{Rec}}_{i}|$, and $|\mathrm{Non}\text{-}{\mathrm{robust}}_{i}|$. We also list $|Y_{i}^{\mathrm{end}}|$ (after deduplicating multiple successors produced by the same terminal event at the same global state). For instance, in $G_{4}$, all paths to the single terminal state are via unreliable events; hence, $|{\mathrm{Strict}}_{4}\left|=\right|{\mathrm{Rec}}_{4}|=0$ and $|{\mathrm{Non}\text{-}\mathrm{robust}}_{4}\left|=\right|Y_{4}|$. The results in Table 9 are consistent with this semantics.

Optional, sensitivity to unreliable events: We vary the fraction *p* of events marked as unreliable and recompute the labels using the same product–events terminals. A simple trend plot can show how increasing unreliability reduces $|{\mathrm{Rec}}_{i}|$ and increases $|Non\text{-}robust_{i}|$.

Takeaway. The centralized reachable graph is an order of magnitude larger than the sum of locals (Table 7), whereas our detection cost scales linearly in $n_{i}+m_{i}$ on each $G_{i}$ (Table 8). The proposed distributed scheme therefore avoids constructing the global synchronous product at runtime and scales structurally with the sum of local sizes. This substantiates the claims on modularity and scalability without relying on platform-dependent wall-clock timing.

## 6. Conclusions

In conclusion, the proposed modular and distributed supervisory control integration framework provides a scalable and robust solution for automated and micro-manufacturing systems experiencing event-level failures. The method’s compatibility with industrial information integration standards, micro-fabrication platforms, MEMS-oriented production environments, and edge control infrastructures makes it highly suitable for deployment in real-world smart and micro-manufacturing applications. Simulation studies confirm its effectiveness in maintaining system robustness and adaptability while avoiding unsafe trajectories. Future work will focus on practical deployment and integration within industrial and micro-manufacturing execution systems, as well as experimental validation on MEMS-based devices, industrial edge controllers, and cloud-based control infrastructures. To facilitate deployment, we also distill two practice-oriented points clarified in this revision-online update of unreliable events and the applicability beyond full local controllability/observability.

Online update of unreliable events: The detector graph does not depend on the reliable/unreliable split; only the labels produced by Algorithm 1 do. Therefore, if the set $E_{ur}$ changes at runtime, each affected subsystem can recompute labels locally in linear time $O(|Y_{i}|+|\to_{i}|)$ or hot-swap one of a few precomputed label tables for anticipated modes (e.g., a ‘resource-unreliable’ flag). The update is safety-monotone: declaring more events as unreliable can only shrink the enabled set and thus preserves safety while possibly becoming more conservative until a reliable suffix exists.

Finally, we note that the above study is carried out under a simplifying assumption of full local controllability and observability; the paragraph below outlines how the framework can be relaxed when this assumption is violated.

Limitations and extensions beyond full controllability/observability: The results above rely on full local controllability and observability. When some events are uncontrollable or unobservable, Algorithm 1 can be adapted as follows:

- Limited control: Replace the reliable label set by the enforceable set $E_{i}^{\mathrm{enf}}:=E_{i}^{\mathrm{rel}}\cap E_{i}^{c}$. Then, in Step 3 (ForwardClosureRestricted on reliable labels), use $E_{i}^{\mathrm{enf}}$ instead of $E_{i}^{\mathrm{rel}}$. This ensures that Strict states admit a reliable and controllable prefix inside $B_{\mathrm{rel}}$.
- Partial observation: Build an observer (or belief-state) automaton over the observable alphabet $E_{i}^{o}$ (or equivalently run Algorithm 1 on the fly over observed state sets using the projection $P_{i}$). This yields labels consistent with what supervisors can infer from observations.
- Mixed case: Combine the two by running Algorithm 1 on the observer with enforceable labels $E_{i}^{\mathrm{enf}}$.

A rigorous development, together with complexity/approximation techniques to mitigate observer blow-up, is left as future work.

## Figures and Tables

**Table 1:** Robustness-classified detector states $Y_{D_{1}}$ of subsystem $G_{1}$.

State	Component Markings (Product + Resources)
$y_{0}^{\left(1\right)}$	$\left(x_{0},x_{13},x_{14}\right)$
$y_{1}^{\left(1\right)}$	$\left(x_{1},x_{1},x_{14}\right)$
$y_{2}^{\left(1\right)}$	$\left(x_{0},x_{4},x_{14}\right)$
$y_{3}^{\left(1\right)}$	$\left(x_{0},x_{7},x_{7}\right)$
$y_{4}^{\left(1\right)}$	$\left(x_{2},x_{13},x_{2}\right)$
$y_{5}^{\left(1\right)}$	$\left(x_{0},x_{13},x_{5}\right)$
$y_{6}^{\left(1\right)}$	$\left(x_{0},x_{8},x_{8}\right)$
$y_{7}^{\left(1\right)}$	$\left(x_{2},x_{4},x_{2}\right)$
$y_{8}^{\left(1\right)}$	$\left(x_{1},x_{1},x_{5}\right)$
$y_{9}^{\left(1\right)}$	$\left(x_{0},x_{4},x_{5}\right)$
$y_{10}^{\left(1\right)}$	$\left(x_{0},x_{9},x_{14}\right)$

**Table 2:** Reliable-event enabling rule induced by the local detector.

Robustness Label	Enabled Events at State $y_{i}$
Strict	$\left\{\;e\in E_{i}|Post_{e}\left(y_{i}\right)\subseteq {\mathrm{Strict}}_{i}\;\right\}$
Recoverable	$\left\{\;e\in E_{i}|Post_{e}\left(y_{i}\right)\subseteq {\mathrm{Rec}}_{i}\;\right\}$
Non-robust	*⌀*

**Table 3:** Mapping of unreliable events to typical micro-manufacturing faults.

Unreliable Event	Physical Fault in Micro-Manufacturing
$e_{9}$	Tool jam/stage over-current
$e_{10}$	Misalignment detected by vision
$e_{12}$	Loss of vacuum/grip failure
$e_{13}$	Vision reject after inspection

**Table 4:** Robustness classification summary for each subsystem.

Subsystem	Total States	Strictly Robust	Non-Robust
G1	11	8	3
G2	11	8	3
G3	>30	mixed	3+
G4	5	0	5

**Table 5:** Centralized vs. distributed structural performance comparison.

Metric	Centralized	Distributed	Observation
Reachable States	10	11 + 11 = 22	Distributed slightly larger
Supervisor Reusability	No (monolithic)	Yes (modular)	Enables reuse
Incremental Integration	No	Yes	Distributed supports local addition
Supports $G_{3}$, $G_{4}$ Extension	No	Yes	Distributed scalable to larger systems

**Table 6:** Qualitative comparison between centralized, static FTC, modular supervision, and the proposed distributed robust supervision.

Feature	Centralized	Static FTC	Modular Sup.	Proposed (Dist. Robust)
Unreliable Event Handling	✗	✓ (predefined)	✗	✓ (dynamic)
Recoverable Robustness	✓	✗	✗	✓
State-Space Scalability	✗	✓	✓	✓
Supervisor Reusability	✗	✗	✓	✓
Incremental Integration	✗	✗	✓	✓

**Table 7:** Structural size comparison: centralized reachable graph vs. sum of locals.

	Centralized |X|	Centralized |⇒|	$\sum_{i}n_{i}$	$\sum_{i}m_{i}$
Counts	**26**	**60**	**69**	**151**
Explosion factors	${\mathrm{EF}}_{\mathrm{states}}=0.377$	${\mathrm{EF}}_{\mathrm{edges}}=0.397$		

**Table 8:** Per-automaton structural workload (edge visits) and peak queues.

*i*	$n_{i}$	$m_{i}$	$|B_{\mathrm{rel}}|$	$c_{i}^{\mathrm{back}}$	$c_{i}^{\mathrm{all}}$	$c_{i}^{\mathrm{rel}}$	$q_{i}^{\max}$
1	11	16	8	12	12	12	7
2	11	16	8	12	12	12	7
3	42	113	37	75	102	38	27
4	5	6	0	0	0	0	6
∑	69	151	53	99	126	62	37

**Table 9:** Per-automaton label counts (Strict ⊆ Rec) and terminal set sizes.

*i*	$|{\mathrm{Strict}}_{i}|$	$|{\mathrm{Rec}}_{i}\setminus {\mathrm{Strict}}_{i}|$	$|{\mathrm{Rec}}_{i}|$	$|\mathrm{Non}\text{-}{\mathrm{robust}}_{i}|$	$|Y_{i}^{\mathrm{end}}|$
1	8	0	8	8	3
2	8	0	8	8	3
3	19	18	37	37	26
4	0	0	0	5	1
∑	35	18	53	53	33

## Data Availability

The original contributions presented in the study are included in the article, further inquiries can be directed to the corresponding author.
